# Composite correction of a unilateral cleft lip nose deformity and alveolar bone grafting

**DOI:** 10.4103/0970-0358.57190

**Published:** 2009-10

**Authors:** Nitin J. Mokal, Prabhash, Chintamani Kale

**Affiliations:** Department of Plastic and Reconstructive Surgery, Grant Medical College and G. T. Hospital, Mumbai, India

**Keywords:** Alveolar bone graft, Columellar strut, V–Y advancement

## Abstract

**Background:**

Managing the cleft lip nasal deformity has always been a challenge. Even now, there is no single established universally accepted method of correction. The open alveolar gap and the ipsilateral hypoplastic maxilla are two major problems in achieving consistently good results in a cleft lip nasal deformity. In our study, after first assuring the orthodontic realignment of maxillary arches, we combined bone grafting in the alveolar gap and along the pyriform margin, with a formal open rhinoplasty approach.

**Methods:**

All the patients underwent orthodontic treatment for preparation of the alveolar bone grafting. During the process of alveolar bone graft, a strip of septal cartilage graft was harvested from the lower border of the septum which also helps to correct the septal deviation. The cancellous bone graft harvested from the iliac crest was used to fill the alveolar gap and placed along the pyriform margin to gain symmetry. Through open rhinoplasty along the alar rim and additionally using Potter's incision extending to the lateral vestibule, the lateral crura of the alar cartilage on the cleft side was released from its lateral attachment and advanced medially as a chondromucosal flap in a V–Y fashion, in order to bring the cleft-side alar cartilage into a normal symmetric position. The harvested septal cartilage graft was used as a columellar strut. The cleft nostril sill was narrowed by a Y–V advancement at the alar base and any overhanging alar rim skin was carefully excised to achieve symmetry.

**Results:**

The results of this composite approach were encouraging in our series of 15 patients with no additional morbidity and a better symmetry of the nose and airway especially in the adolescent age group.

**Conclusion:**

This concept of simultaneous approach when appropriate for nasal correction at the time of alveolar bone grafting showed an encouraging aesthetic and functional outcome.

## INTRODUCTION

Cleft lip nasal deformity offers a unique challenge to the reconstructive surgeon but often leaves patients unhappy with residual nasal deformities. As Kernahan[1] aptly stated, ‘a repaired cleft is revealed more by the associated nasal deformity than by the repair line’. Cleft lip nasal deformity becomes a distinct challenge due to various reasons: (1) the clinical presentation varies widely, requiring a host of surgical techniques. (2) The deformity may be quite asymmetric, making correction difficult. (3) Patients with a cleft lip might have undergone several previous operations leading to significant scarring. (4) The timing of rhinoplasty whether staged or synchronous with a cleft lip repair is still a subject for debate. (5) As this anomaly affects the paediatric population, the effect of and on patients' facial growth needs to be considered before nose correction. The complexity of the problem is reflected by the fact that there are possibly as many methods to correct these deformities as there are surgeons performing operations.[2–10]

The basic objectives of nasal surgery however are fairly well defined:

1. To restore the symmetry of the maxillary support, alar cartilages and septum.[8–10]
2. To produce a cosmetically acceptable nasal tip.
3. To obtain an optimum functional unit as an airway and humidifying organ.
4. To obtain a satisfactory relationship between the lip and nose.

It must be emphasized that there is no single method or manoeuvre for the correction of the cleft lip nasal deformity which is satisfactory for every patient. It is therefore essential to be familiar with various methods of repair and to tailor them singly or in combination as required in any particular case.

## MATERIALS AND METHODS

Patients having an alveolar gap were chosen for composite correction, i.e. both alveolar and pyriform margin bone grafting and secondary rhinoplasty. All patients were sent for an orthodontic arch alignment.[11,12] If primary lip repair was considered inadequate, it was revised at the same time. Our approach to the patient involved the following modalities.

### Evaluation

Each case was carefully evaluated preoperatively and the nasal deformity was analysed. Attention was paid to the deformities like, alveolar arch alignment, nostril differences, position and level of the alar base, columellar length and the presence of a vestibular and alar web.

### Plan

**Figure 3:**
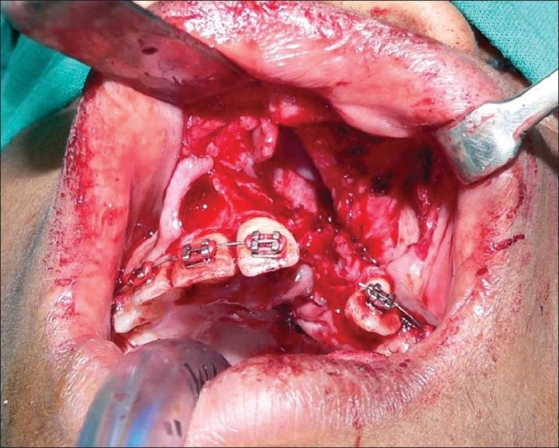
Alveolar defect with suturing of nasal & oral lining ready to accept cancellous bone graft

**Figure 4:**
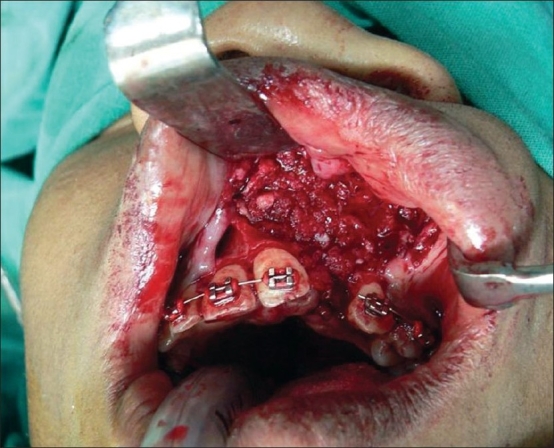
Cancellous bone graft to fill in the alveolar defect and pyriform area
- **For alar cartilage asymmetry**
  An open rhinoplasty approach was taken to expose the cartilage framework[13] [Figure 5]. Cleft-side alar cartilage was raised as a chondromucosal flap and shifted upwards and medially in a V–Y fashion[13–15] [Figure 6]. The harvested septal cartilage graft was placed between the medial crurae of alar cartilages next to the caudal border of the septum as a columellar strut [Figure 7]. The dome of the cleft-side alar cartilage was sutured to the dome of the opposite side with the suture also passing through the tip of the septal graft to achieve necessary tip projection, symmetry and support[16] [Figure 8].

Depending upon the severity of the deformity, a surgical plan was decided. The remedy of each deformity was standardized as far as possible and is briefly elaborated below:

- **For alveolar gap and maxillary platform asymmetry with a depressed alar base**
  Gingivoperiosteal flaps were sutured on the palatal side and nasal mucoperiosteal flaps from the pyriform area sutured with the vomerine mucoperichondrial flap to repair the nasal floor [Figure 3]. This three-dimensional defect was then filled with cancellous bone graft from the iliac crest. Additional bone graft was placed along the pyriform margin[8,11,12] [Figure 4].

**Figure 5:**
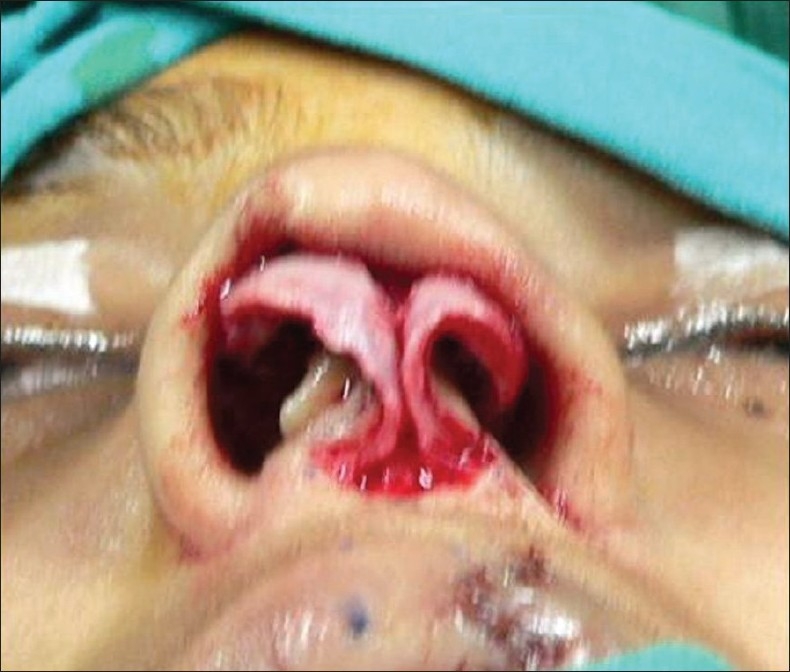
Open rhinoplasty exposing both alar cartilage, showing asymmetry

**Figure 6:**
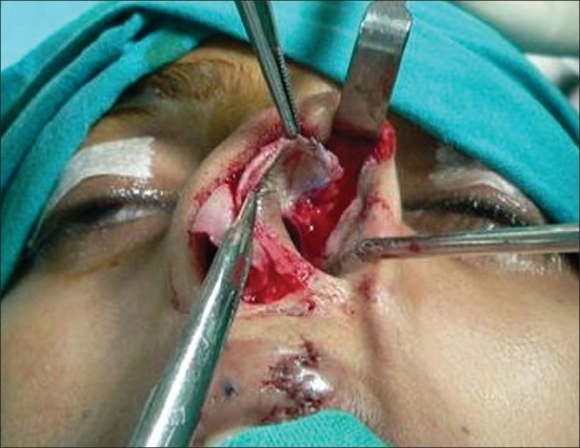
Chondromucosal V-Y advancement of lateral crus of alar cartilage to reach the level of opposite alar cartilage

**Figure 7:**
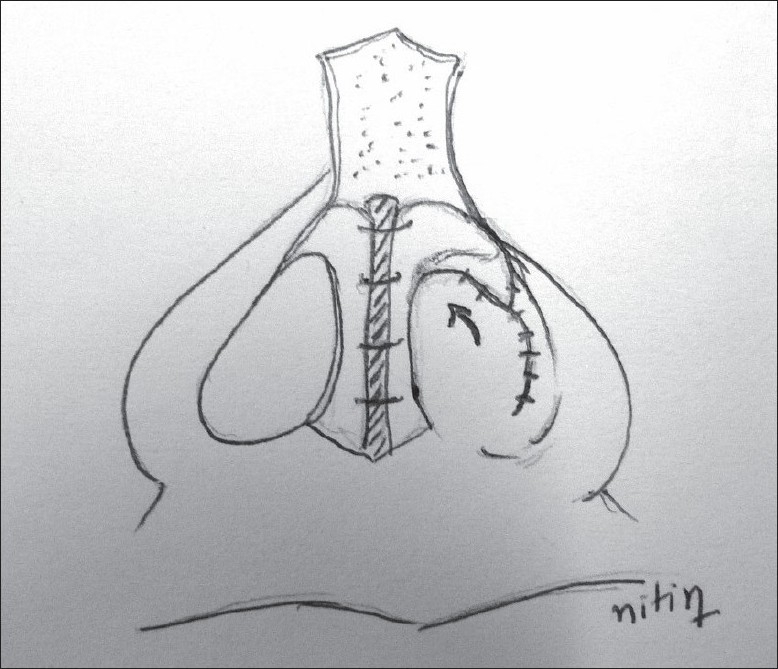
Diagram showing the placement of the columellar strut and closure of V defect to Y after de-bulking the soft tissue in that area

**Figure 8:**
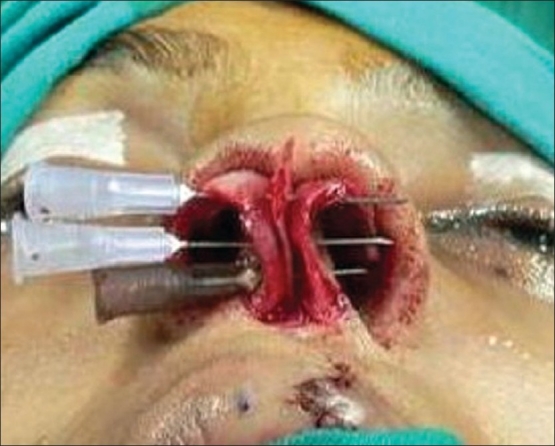
Columellar strut septal graft placed between alar cartilages and fixed in symmetric and elevated position
- **For the vestibular web**
  The lateral process of the alar cartilages on the cleft side was released from its lateral attachment and advanced medially as a chondromucosal flap in a V–Y fashion. Debulking of the fibro-fatty tissue allowed easy closure and obliteration of the vestibular web.
- **For unequal nostril sill**
  A Y–V advancement of the alar base medially was performed and an alar cinch stitch was taken for narrowing the nostril base.[17]
- **For septal deviation**
  The resection of a strip of the cartilaginous septum along its lower border and re-positioning it in the midline allowed correction of the septal deviation.[9]
- **For tip projection:** The resected part of the lower border of septum was used as the columellar strut and the alar cartilages were fixed to the strut.[16]
- **For the alar web**
  Alar web excision was performed. It apparently improved the length of the columella on the cleft side[2,18] [Figures 9 and 10].

**Figure 9:**
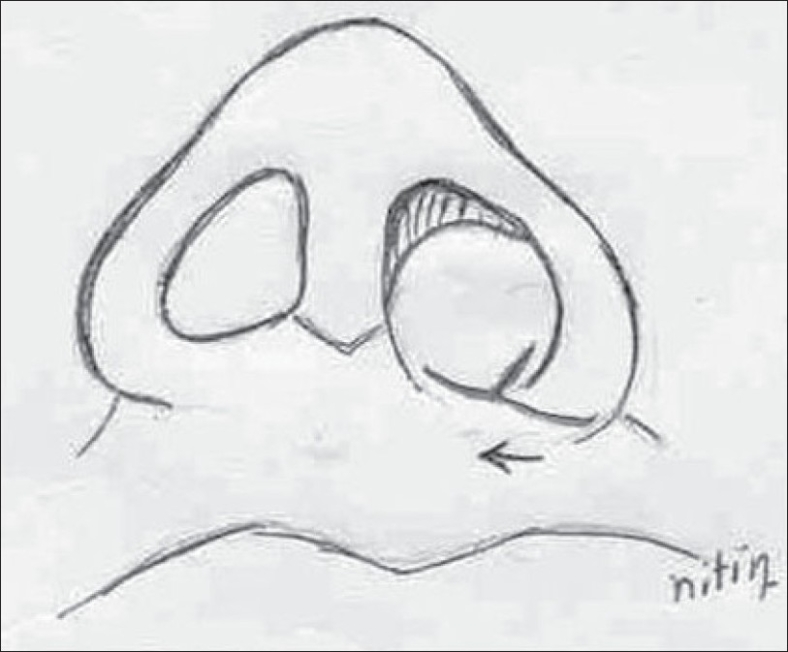
Diagram showing Y-V alar base advancement for nostril floor narrowing and area of overhanging alar web needs excision

**Figure 10:**
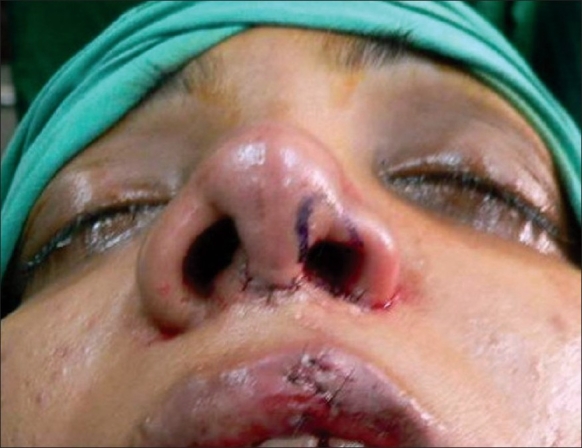
Marking of the alar web excision after Y–V alar base narrowing

### Procedure

**Figure 1:**
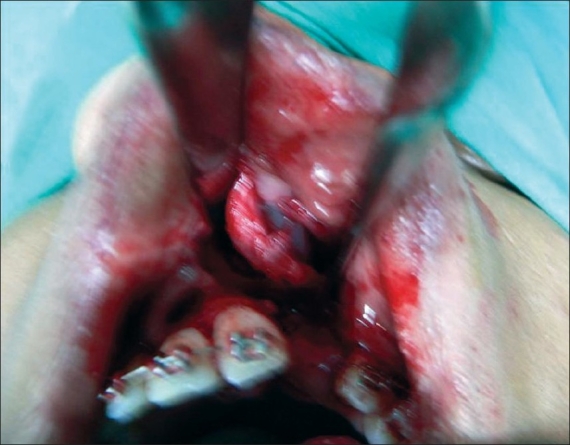
Exposure of the lower border of the septum through alveolar gap

**Figure 2:**
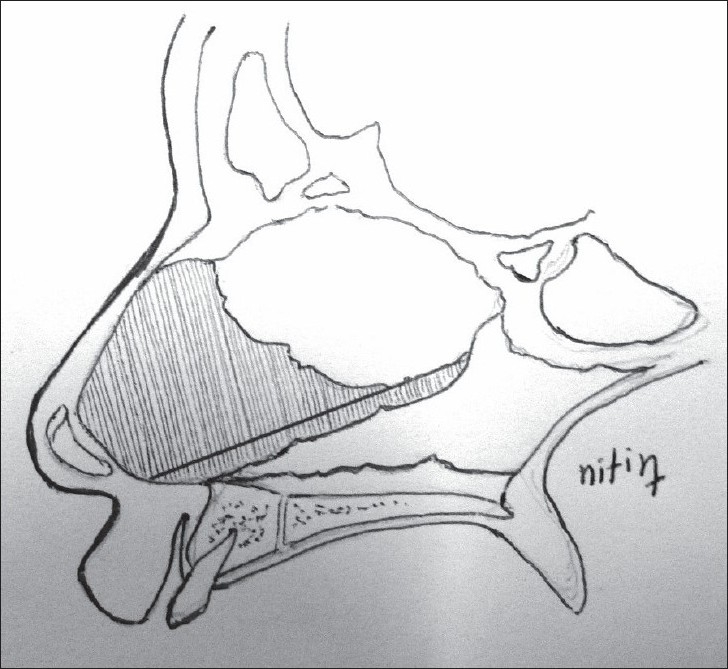
Diagram showing area of septal graft harvest

We start the procedure with the alveolar bone grafting. Incisions are made along the alveolar cleft margin and gingivoperiosteal and mucoperiosteal flaps are raised along the nasal and buccal surfaces. The base of the septum is exposed through the same approach and sub-mucoperichondrial dissection is done on both sides of the septal cartilage to expose and identify the attachment of the septum to the palatine crest. A strip of cartilage is harvested from the lowermost part which helps in the correction of septal deviation [Figures 1 and 2]. The nostril floor is repaired using a superiorly based mucoperichondrial layer of the vomer flap and nasal mucoperiosteal lining along the pyriform margin. Palatal gingivoperiosteal layers are sutured together to complete the oral lining [Figure 3]. Cancellous bone graft is harvested from the iliac crest and filled in the three-dimensional bony defect in the alveolus [Figure 4]. Additional bone graft is added along the pyriform margin. A water-tight mucoperiosteal closure is achieved.

For the open rhinoplasty, a Potter's incision[13] is made from the lip columella junction, extending to the caudal margin of both alar cartilages. The skin envelope is separated widely from alar cartilages and upper lateral cartilages on both the cleft and non-cleft sides [Figure 5]. On the cleft side, a chondromucosal flap of the lateral crura of the alar cartilage is raised and advanced medially in a V-Y fashion [Figure 6]. Dissection is done between the two alar cartilages anteriorly and the caudal border of the septum is identified.

The base of the septal cartilage is mobilized and fixed to the periosteum of the anterior nasal spine using non-absorbable sutures. The piece of cartilage which was harvested from the lower border of the septum is now placed between two medial crura of the alar cartilages anterior to the caudal border of the cartilaginous septum. The medial crus of both alar cartilages is fixed to this cartilaginous strut with 5-0 PDS sutures and this complex of alar cartilage and strut is fixed to the caudal border of the septum using absorbable 5-0 PDS sutures [Figures 7 and 8]. Additional inter-domal stitches are passed between the two alar cartilages and the cartilage graft to produce appropriate tip projection. The supero-medial advancement of the V-shaped chondromucosal flap of the lateral crura on the cleft side leaves a defect in the nasal lining which is then closed in a Y shape after debulking of the fibro-fatty tissue. This also helps in the correction of the vestibular web. Asymmetry of nostril size is assessed and a medial Y–V advancement of the alar base is done along with an alar cinch stitch on cleft side to narrow the nostril and to achieve symmetry with the opposite side [Figure 9].

For the correction of the residual alar web, an assessment is made of the overhanging skin in the soft triangle area. This is then marked and excised very carefully from medial to lateral, keeping its lateral attachment intact to be excised only at the end depending on the requirement of the skin to fill the defect caused by the movement of the chondromucosal flap of the lateral crus mentioned earlier [Figure 10]. The open rhinoplasty incision is then closed all along using a 4-0 or 5-0 chromic catgut.

Intranasal packing is done using paraffin gauze. Sterile paper tapes are applied over the dorsum to facilitate the re-draping of the skin envelope to the cartilaginous framework. Cold compresses are advised during the post-operative period to reduce oedema formation. Besides a single parentral preoperative antibiotoc, a short course of oral antibiotics is prescribed for 3 days post-operatively. Intra-nasal packing is removed after 48 h.

### Post-operative assessment

The post-operative assessment of results was done subjectively, as is the usual practice, by asking a panel of five adults to look at only the post-operative photographs in all three views (frontal, oblique and lateral) and grade the result into three categories: good, satisfactory and poor. The panel consisted of five members, four of whom had no knowledge of plastic surgery and the remaining member was the author himself.

After the panel had graded the results, they were shown the pre-operative photographs and were asked to judge the post-operative results again. The purpose of this was to determine whether one's subjective assessment of the result was influenced by the improvement achieved by surgery.

While judging results, the axis of nose, and its deviation from the midline (vertical line passing through the mid-canthal point), if any, symmetry of both ala, tip projection, projection of the dorsum and supra-tip in relation to the tip and symmetry of the bony dorsum and especially the position of the septum in relation to the midline were kept in mind by the author[19,20] [Figures 11–20].

**Figure 11:**
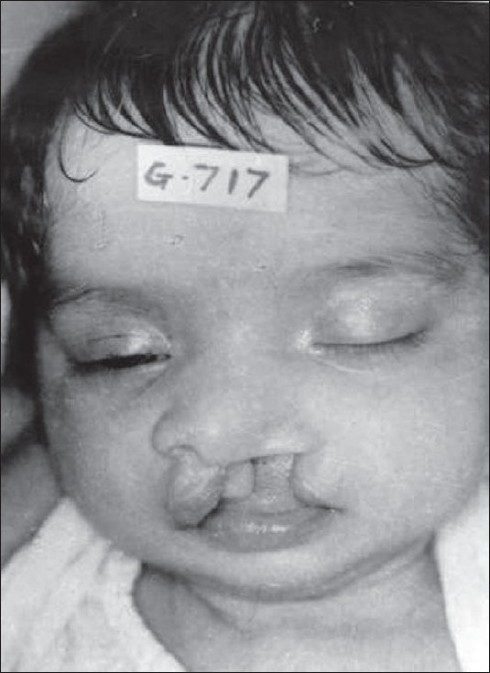
3 month old baby with cleft lip and palate

**Figure 12:**
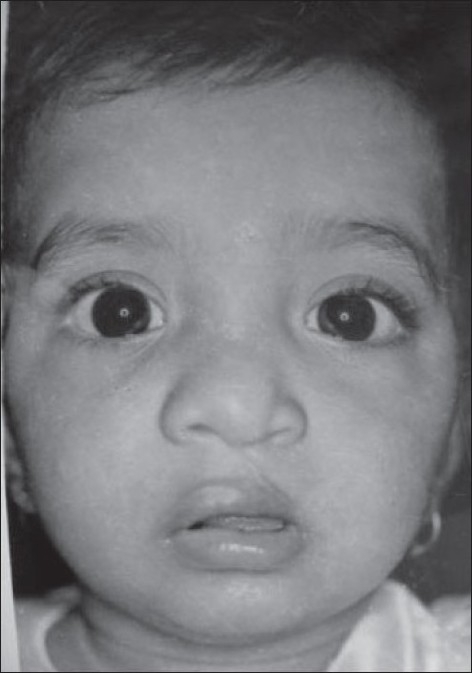
One year post operative view

**Figure 13:**
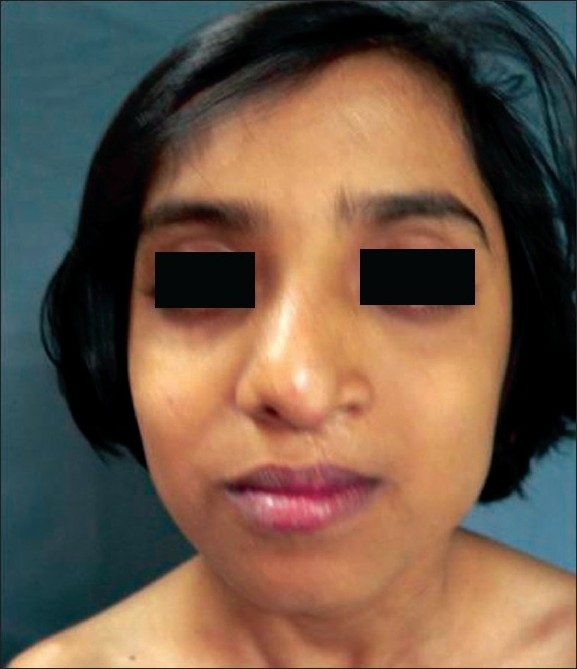
The same 9 year old girl with classic cleft nose deformity

**Figure 14:**
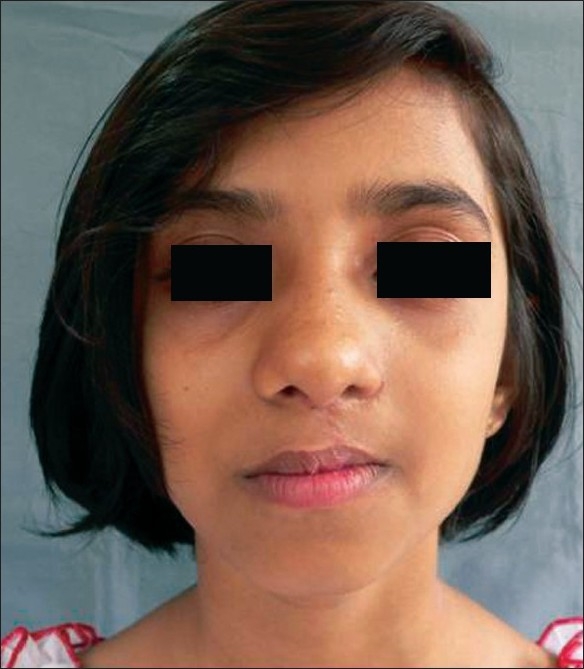
Post op result with symmetry

**Figure 15:**
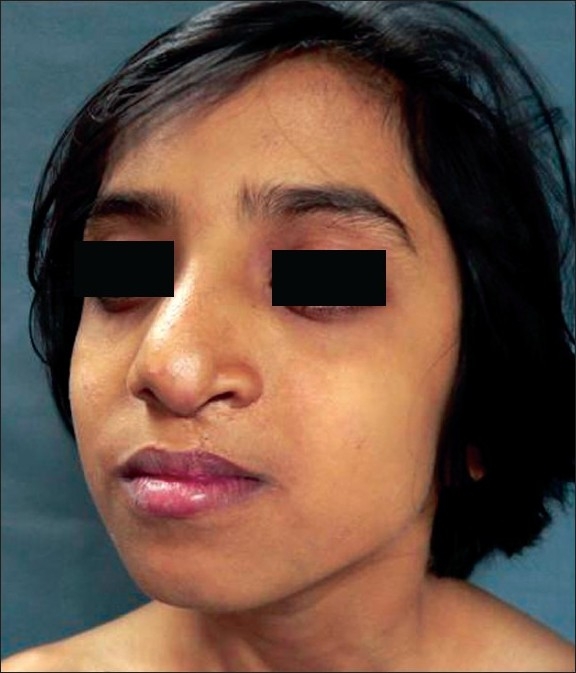
Same 9 yr old girl in profile showing drooping alar cartilage

**Figure 16:**
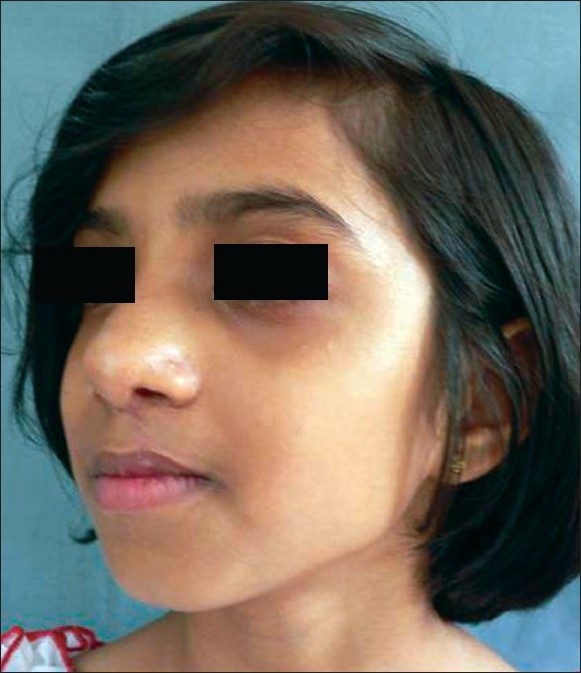
Post op result showing restoration of nasal tip projection

**Figure 17:**
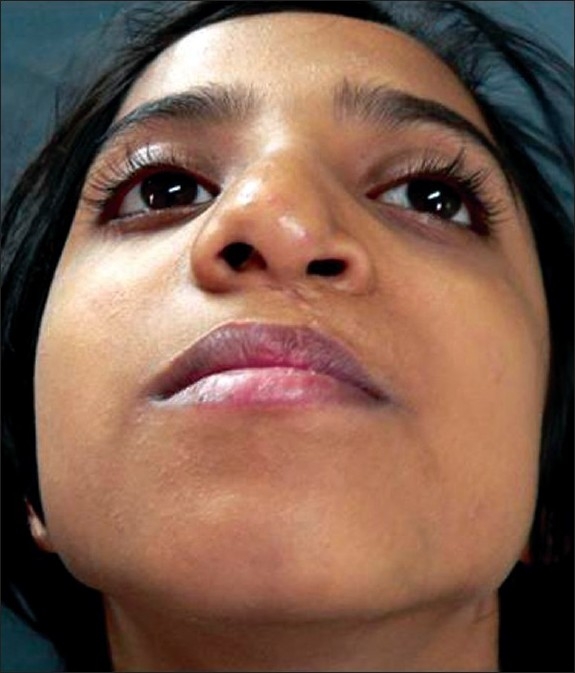
Worm's eye view showing drooping of alar cartilage

**Figure 18:**
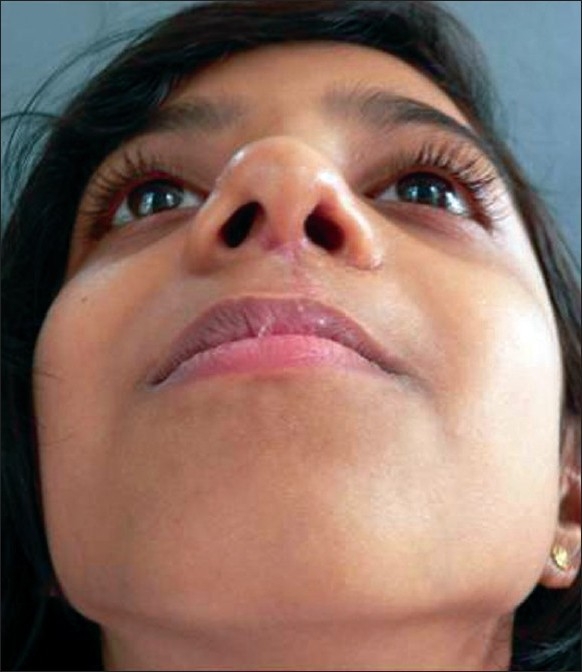
Post op result with symmetry and projection

**Figure 19:**
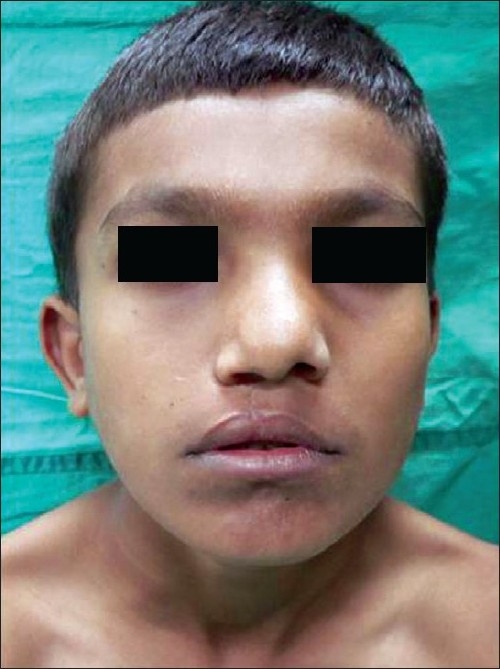
11 yr old boy with classic cleft nose deformity

**Figure 20:**
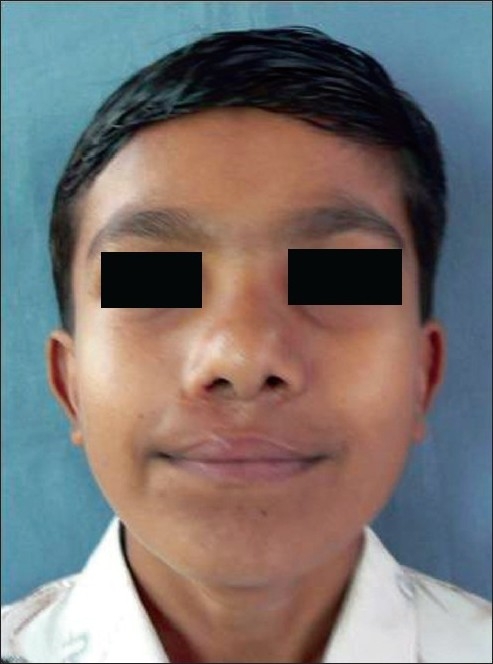
Post op result with symmetry

Besides the above, the patient was asked about the air flow through both nostrils while breathing normally and during forced breathing to assess the effect of septal correction.

## DISCUSSION

The history of secondary correction of the cleft nose deformity starts with external incisions with or without rotational advancements, incision approaches to tip, repositioning of alar cartilages and cartilage grafts to shape and maintain the tip. Use of an external incision encompassing the length of the columella, extending up to the tip and even a flying-bird incision are fading into obscurity as wide exposure can be readily obtained by bilateral rim incisions that may be connected by a transverse incision at the base of the columella[13] or by stair-step incision at the mid-columella.

We focused our attention on filling the alveolar bone defect and obtaining the symmetry of the nose in all its aspects including both bony and cartilaginous parts and the base. It is already clear that no single procedure can achieve all these; so the individual deformities were fully analysed and a corrective plan was made according to the need.

We found that simultaneous alveolar bone grafting does not cause any additional morbidity. In fact the bone graft placed along the pyriform margin dramatically improves the results of nasal correction.

Raising of a mucoperichondrial vomer flap needed for the closure of the nasal lining of the alveolar gap provides access to the septum to harvest a cartilage graft and correct its deviation simultaneously.

We found Potter's method[13] to be most useful as it allows correction in entirety. This open rhinoplasty incision provides good exposure for the correction of cartilages under vision. The columellar scar lies in a natural columella-labial crease rather than in mid-columella, which may produce contracture and narrowing later on. It can easily be combined with lip and nostril revision procedures and there is no risk of inadvertent transection of cartilages. Re-draping of skin over a modified cartilage framework is never a problem.

It has been reported that this method has a disadvantage that due to cutting of lateral ligaments, it can produce valving and a cosmetic depression in this region.[8] In our series, we do not leave behind any intranasal raw area whatsoever and hence we have not found any such deformity in our study.

This holistic approach for the treatment of this multidimensional deformity has gained support and acceptance by many surgeons like Salyer and Cutting[8,9] in the past. The cleft lip nasal deformity is a complex three-dimensional problem. The plethora of techniques described in the literature is clearly indicative that no single method can correct all the components of the deformity and combinations of procedures should be used for the best possible result. The operating surgeon should focus not only on the aesthetic appearance of the nose but also its function as an airway.

Alveolar bone grafting can be safely and effectively combined with secondary unilateral cleft lip rhinoplasty. This along with a bone graft along the pyriform margin improves symmetry and provides an aesthetically pleasing nose without any additional morbidity.

Studies involving a larger patient group and longer follow-ups can significantly change our concepts and the management of this multidimensional problem. The results of our procedure were encouraging in our 15 consecutive patients with no additional morbidity and better appearance of nose correction especially in the adolescent age group.
